# Designing and evaluating an interprofessional education conference approach to antimicrobial education

**DOI:** 10.1186/s12909-020-02252-9

**Published:** 2020-10-13

**Authors:** Clare Guilding, Jessica Hardisty, Elsa Randles, Louise Statham, Alan Green, Roshni Bhudia, Charan Singh Thandi, Andrew Teodorczuk, Lesley Scott, Joanna Matthan

**Affiliations:** 1 School of Medical Education, Faculty of Medical Sciences, Newcastle University, Framlington Place, Newcastle Upon Tyne, NE24HH UK; 2 Sunderland Pharmacy School, University of Sunderland, Sunderland, UK; 3 School of Medicine, Griffith University, Gold Coast, Queensland Australia; 4 The Prince Charles Hospital, Metro North Mental Health, Brisbane, Queensland Australia; 5 School of Nursing and Health Science, University of Sunderland, Sunderland, UK; 6 School of Dental Sciences, Faculty of Medical Sciences, Newcastle University, Newcastle Upon Tyne, UK

**Keywords:** Interprofessional education, IPE, Interprofessional learning, Simulation, Prescribing, Antibiotics, Antimicrobial stewardship, Human errors, Pharmacy, Medicine

## Abstract

**Background:**

Arguably, Medical School curricula are deficient in learning opportunities related to the safe and effective use of medicines, in particular antimicrobials. Infection management is complex and multidisciplinary, and learning opportunities should reflect these principles. Aligned to the complexity of the subject matter, simulation and interprofessional based teaching are methods that can foster the collaborative skills required of future healthcare professionals. There have been calls to develop these methods in the teaching of safe prescribing and the management of infections; however, reports of such studies are limited.

**Methods:**

We developed an interprofessional education (IPE) conference for second year undergraduate medical and pharmacy students based in the North East of England. We considered contact theory in the design of three small group interprofessional workshops, on the broad themes of antimicrobial stewardship, infection management and patient safety. A mixed methods approach assessed students’ attitudes towards IPE, barriers and facilitators of learning, and perceived learning gains. Qualitative data from workshop evaluation forms were analysed thematically, while quantitative data were analysed descriptively and differences between medical and pharmacy cohorts analysed using unpaired two-tailed t-tests.

**Results:**

226/352 students returned the workshop evaluation forms (66% of pharmacy students, 62% of medical students). 281/352 students responded to a series of Likert scale questions on the value of interprofessional education (88% of pharmacy students, 70% of medical students). Students reported acquisition of knowledge and skills, including concepts and procedures related to infection management and antimicrobial prescribing, and the development of problem-solving and critical evaluation skills. Students reflected on their attitude towards interprofessional collaboration. They reported a greater understanding of the roles of other healthcare professionals, reflected on the importance of effective communication in ensuring patient safety, and were more confident to work in interprofessional teams after the conference.

**Conclusions:**

A robust IPE event, theoretically underpinned by contact theory and developed collaboratively, achieved interprofessional learning at scale and helped develop healthcare professionals willing to collaborate across disciplines. The resources, and evaluation insights based on the 3P (presage, process, and product) model of learning and teaching, will be of value to other educators who seek to develop theoretically-sound IPE interventions.

## Background

The necessity for antimicrobial stewardship to be embedded into pre-qualification teaching for healthcare professionals is well established, with emphasis on how antimicrobial resistance is prevented and managed [1]. Appropriate prescribing of antimicrobials requires high-level clinical and diagnostic reasoning skills in order to determine whether such treatment is indicated and in choosing a suitable agent to reduce the risk of sepsis and other complications [2]. The focus on reducing the use of antimicrobials should be balanced against an ability to recognise when prescribing is clinically indicated.

Studies exploring how antimicrobial prescribing and stewardship are embedded in undergraduate (UG) medical, dental, pharmacy, physician associate, nursing, midwifery and allied health professional courses demonstrate that, although included in most degrees, the depth of, and number of principles covered, varied considerably [3, 4]. Current pre-qualification or UG medical provision may not adequately prepare students to prescribe antimicrobials [5]. Junior doctors, reflecting on their practice, reported that they are required to make complicated antimicrobial prescribing decisions in challenging working environments with insufficient preparation from their UG training and conflicting information provided by colleagues or senior staff [6]. Similarly, variability across pharmacy schools with regards to the taught content around antimicrobial stewardship has been found, and pharmacy students and educators have reported that additional training is required [7].

More broadly, the preparedness of medical school graduates in the UK to take on their prescribing role on entering clinical practice has been mixed [8, 9]. Current evidence suggests that junior doctors lack the clinical pharmacology and therapeutics knowledge required on graduation [8, 10, 11]. The EQUIP study, which provided an in-depth exploration of the experiences of medical students and junior doctors, highlighted a lack of learning opportunities related to safe and effective use of medicines [12]. 8.9% of all hospital prescriptions in their study had prescription errors, with junior doctors showing the highest error rates (8.4 and 10.3% for Foundation Year 1 and 2 respectively). The class of medication with most prescription errors was antimicrobials, associated ‘with a median error prevalence of 32% of orders’ [12]. Pharmacists and nurses intercepted almost all serious errors before they caused harm, but in some cases were themselves the cause of error, highlighting the complexity around human errors in prescribing [12]. Thus, these challenges are not limited to medical prescribers. Moreover, with increasing prescribing roles emerging for allied healthcare professionals, such as physician associates, pharmacists and nurses, it is important that the entire multi-professional clinical team receive sufficient targeted and tailored education and training to develop knowledge and skills in this key area.

### Interprofessional education approach to antimicrobial prescribing

Interprofessional education (IPE), which occurs when ‘two or more professions learn with, from and about each other to improve collaboration and quality of care’ [13], is an approach recommended for improving prescribing practice [12, 14]. At a theoretical level, given the complexity of prescribing decisions and the need for input from all professions, IPE as a strategy to build collaborative practice is crucial as a coherent educational approach. Indeed, the provision of opportunities for IPE was a key recommendation of the EQUIP study [12]. Other recommendations were to teach and assess practical prescribing in all pre-qualification programmes, with prominence given to commonly prescribed drugs such as antimicrobials. In response to reports highlighting the impact of poor prescribing on antimicrobial resistance, the UK Specialist Advisory Committee on Antimicrobial Resistance advocated ‘a coherent multi-disciplinary approach to the entire process of antimicrobial prescribing’, grounded in IPE [14].

There is evidence that incorporation of IPE in healthcare curricula produces health and social care graduates whose attitudes are more aligned with those required for effective multidisciplinary working, and that it is a valuable methodology to employ when delivering learning and teaching around prescribing skills and medication safety [15–18]. Simulation-based-learning is an integral part of many IPE programmes and indeed it has been argued that IPE where possible should be coupled directly with simulation [19]. High-fidelity simulations can replicate the complexities and stresses of the clinical working environment and facilitate development of team-working and collaboration skills [20–22]. In Medical School education, high-fidelity simulations are most frequently targeted at students in the transition into clinical practice [23, 24]. Only a few examples exist of high-fidelity simulation for early years’ clinical pharmacology/pharmacy education [25, 26] and high-fidelity simulations have yet to adequately tackle safe prescribing [8, 10, 11].

### Aims and objectives

This study aims to design and evaluate an IPE conference to inform future developments of interprofessional antimicrobial teaching activities within and outwith the North East of England. The objectives of this study were to explore:

i. the use of contact theory in the design of IPE activities
ii. the perceived acquisition of knowledge and skills from the IPE conference workshops
iii. the facilitators and barriers to learning in each IPE conference workshop, and suggestions for change
iv. the utility of the conference in promoting the value of collaborative practice.

## Methods

### Context and theoretical basis for undergraduate pharmacy-medicine IPE

The IPE conference was developed jointly by a cross-institutional steering group of faculty from medicine (MBBS) and pharmacy (MPharm) programmes at two universities in the North East of England. The conference was hosted within the Faculty of Medical Sciences at Newcastle University in 2016. We previously assessed the feasibility and logistics of delivering large scale, cross-institutional IPE in a conference format [27]. This study follows on, to detail the design and evaluation of the individual workshops within the conference.

We used contact theory to guide the design and development of the content. Contact theory, as first proposed by Allport [29], is one of the key theoretical perspectives on IPE [29–32]. Allport hypothesised that positive intergroup interaction, which should reduce stereotyping that hampers interprofessional collaboration, depends not only on bringing the groups together, but on four conditions being met (see Table 1).

**Table 1:** Allport’s four conditions for positive interprofessional interaction [29]

Condition 1	Equal status between the different groups
Condition 2	Groups should work on common goals
Condition 3	Groups should cooperate and not compete with each other
Condition 4	The activities/programme should have support of authorities (e.g. institutional support)

Equal status (condition 1, see Table 1) requires identification and matching of the level of the students. This matching relates to the number of years in education and the level of subject-specific knowledge gained [33]. The learning outcomes (LOs) should be relevant to both programmes with students working on common goals (condition 2, see Table 1). We designed this initiative to enhance education around the management of infections and introduce students to the importance of effective interprofessional working in ensuring patient safety. Collectively, students had covered pharmacology, microbiology, sepsis and antimicrobial stewardship outcomes by Semester 2 of Year 2, their last semester of pre-clinical education. Consequently, this study used Year 2 medical (MBBS) students from Newcastle University and Year 2 pharmacy (MPharm) students from the University of Sunderland.

There were variations in the content previously covered by each cohort. Pharmacy students had previously covered content on practical prescribing and use of the British National Formulary (BNF), while medical students had not. Medical students had covered clinical aspects of the diseases that the cases were based around (e.g., meningitis and sepsis), while the pharmacy students had not. These differences were exploited to design interprofessional tasks that required the combined knowledge and cooperation of both professional groups, where collaborative practice would bridge gaps in learning and facilitate a new mutual understanding (condition 3, see Table 1).

Authority support (condition 4, see Table 1) came from both institutions, together with a range of external stakeholders. The institutions both contributed funding towards the project. National Health Service (NHS) support was showcased in the opening keynote presentations, given by the Regional Advisor for Education - Health Education England North East (HENE) and the local NHS Foundation Trust Chief Pharmacist. Other regulatory and professional body stakeholders including the Centre for Advancement of Interprofessional Education (CAIPE), UK Clinical Pharmacy Association, National Pharmacy Association and British Pharmacological Society contributed stalls on the day.

### Workshop design

Three workshops were designed to cover both explicit and implicit IPE curriculum content, using a variety of educational methods [34]. In an *explicit* approach, IPE itself is the focus of the session, while in an *implicit* approach the core topic is the clinical case, with IPE competencies such as teamwork and communication experienced through the process of completing the cases collaboratively. Correspondingly, LOs were both subject-specific (e.g., ‘*Describe the principles of antibiotic use’*; ‘*Describe the rationale behind the selection of an appropriate antibiotic in meningitis’*) and IPE-specific (e.g., ‘*Explain the importance of collaboration in preventing errors’*; ‘*Understand and value the expertise and values of other team members’*). While the majority of the LOs were already integrated in both curricula and had been taught in previous years in a didactic format, some broader IPE-specific outcomes were added specifically for the conference to guide students into fuller engagement with the programme of the day (see Supplementary File 1, the conference workbook, which includes all outcomes).

Attendance was compulsory, and with approximately 200 students in each programme, workshops activities were designed for balanced numbers of both professions. One hundred ninety-five pharmacy and 157 medical students attended the conference for its pilot run in 2016, and this study presents the data from this pilot. A description of the composition and content of the workshops is shown in Table 2, and the student workbook from the conference can be found in Supplementary File 1. Students were given the cases from the workbook as pre-reading prior to the IPE Conference day.

**Table 2:** The composition and content of the interprofessional workshops

	1. Choosing the Right Antibiotic	2. Significant Event Analysis	3. SimMan Sepsis
Length	2 h	1 h	1 h
Educational approach	Case Based Learning	Video reflections and Significant Event Analysis	High Fidelity Virtual Patient Simulation with Team Based Learning
Student grouping	Mixed groups of up to 6 medical and pharmacy students. 3–4 groups/seminar room.	Mixed groups of up to 6 medical and pharmacy students. 3–4 groups/seminar room.	Mixed groups of up to 8 medical and pharmacy students. 6–7 groups/simulation room.
Facilitators	Doctors and pharmacists together	GP and practicing pharmacist together	Doctors, pharmacists and nurses together
Content	• Two patient cases: Case 1: A patient with a urinary tract infection that developed into pyelonephritis. Case 2: A patient with meningitis. • Students worked together to consider the patients' symptoms and interpret the results of investigations in order to choose the appropriate antimicrobial at each stage of the case and complete prescriptions taking into account dose, duration and route of administration. • In both cases, the patient was initially treated in primary care and then was transferred to secondary care, enabling prescribing practices in each healthcare setting to be discussed.	• A patient safety session focussing on healthcare professional roles, interprofessional communication and error causation. • Featuring the case of a patient with an infection who had received suboptimal care in primary care. Included videos of the healthcare professionals reflecting on the pathway of care and factors that contributed to errors. • Students worked together to complete a significant event analysis, considering the factors that contributed to the development of acute sepsis in the patient. In the case, the patient’s condition deteriorated because of incorrect management and delayed administration of antimicrobials. • Students followed the acute admission of this patient in the SimMan Sepsis workshop.	• A team-based learning exercise using SimMan^3G^ to explore the management of acute sepsis. • Session followed the emergency admission of the patient from the Significant Event Analysis workshop. Students respond to a series of clinical questions to try to treat their patient before he deteriorated. • This required the use of a range of tools, such as the local Antimicrobial Handbook, BNF and the national sepsis management pathway. • Students worked through problems encountered and shared their responses with the entire group through a voting system. • The collective treatment plan was applied to the SimMan so students could observe the real-time effects of their treatment on the virtual patient.

All facilitators underwent training or a masterclass, including a run through of the cases and simulation, emphasising the importance of creating a safe and collaborative learning environment. This was the first formal IPE opportunity both cohorts of students had taken part in within their respective programmes, though students may have had informal interprofessional exposure during clinical placements.

### Data collection

Participants were invited to complete an evaluation form at the end of each workshop. This required them to state three things they learnt from the session, comment on facilitators and barriers to learning, and suggest changes for improvement. Quantitative data assessing students’ views on the value of interprofessional education were collected in a final whole group session, via interactive voting in response to a series of Likert scale questions (see Table 3). The evaluation form and Likert scale questions were developed and refined from previous evaluations of IPE activities at the two universities, and incorporated questions adapted from the Readiness for Interprofessional Learning Scale (RIPLS).

**Table 3:** Student responses to questions on the value of interprofessional education

Statements	Medical (mean ± SD)	Pharmacy (mean ± SD)	***P*** value
The day has made me more confident to work in an interprofessional team	4.12 ± 0.84	4.01 ± 0.98	0.367
The conference helped me understand how the roles of other healthcare professionals contribute to patient care	4.24 ± 0.86	4.01 ± 1.0	0.042*
Shared learning with other healthcare students will help me communicate better with other healthcare professionals	4.42 ± 0.77	4.00 ± 1.1	0.0004*
Shared learning has helped me understand the value and expertise of other professionals	4.37 ± 0.68	3.92 ± 1.0	0.0001*
Shared learning has helped me understand the role and importance of other healthcare professionals in ensuring patient safety	4.33 ± 0.84	4.13 ± 1.0	0.107
I feel ready to learn about the roles of other healthcare professionals at this point in my degree programme	4.13 ± 1.0	3.93 ± 1.2	0.151
I would like to see an increase in interprofessional learning events in the curriculum	4.13 ± 1.0	3.85 ± 1.3	0.059

The mean Likert score for each statement measured using a 5-point Likert scale with 1 = strongly disagree and 5 = strongly agree ± standard deviation for each statement. A two-tail t-test was used to calculate significance (*p < 0.05) between the medical and pharmacy cohorts [35]

We used a convergent parallel mixed methods research design as defined by Creswell & Plano Clark [36]. Data collection was concurrent, followed by separate qualitative and quantitative analysis. Data strands were mixed during interpretation, using theoretical frameworks to bind the datasets (see Discussion). The quantitative data allowed us to determine the students’ general position on the value of IPE and compare responses between cohorts. The qualitative data added a deeper understanding of what, why and how the students have learned.

Ethics was granted by the Faculty of Medical Sciences Ethics Committee at Newcastle University (reference number 4542:2016). All participants were told verbally and in writing that that their consent to participate was voluntary, that the data from the evaluation may be used for publication and they could withdraw their consent and contributions at any time. All participant responses were anonymous, the only distinguishing demographic requested was whether they were medical or pharmacy students. Due to the numbers of students in the sessions, participants were asked to give consent by completing and submitting the evaluation questions.

### Data analysis

Quantitative data collected via the voting handsets were analysed descriptively (mean and standard deviation calculated) and differences between medical and pharmacy cohorts analysed using unpaired two-tailed t-tests (significance set at *p* < 0.05) [35]. For the qualitative data, a three-step inductive (i.e., data driven) thematic analysis, based on the work of Braun and Clarke [28] was used. In this established reflexive approach, coding precedes theme development, with themes subsequently constructed from the codes. Free-text handwritten responses to the questions in the evaluation form were transcribed into Microsoft Excel. The transcribed data were read to identify ‘patterns of meaning and issues of potential interest’ [28], and categorised into broad preliminary codes generated independently by two researchers (RB, CT). Researchers then discussed the two sets of preliminary codes, and combined these to create the agreed final codes for analysis of the data. These two researchers had not been involved with the design and delivery of the conference to minimise potential bias in coding and analysis. In a second step, these codes were analysed, and related codes combined, to generate overarching key themes. Codes and then themes were reviewed and validated by a third researcher (CG). Lastly, each code and theme was quantified to facilitate examination of differences between pharmacy and medical students and differences between the three workshop sessions [37].

## Results

### Quantitative data on the value of interprofessional education

281/352 students responded to a series of statements on the value of interprofessional education (Table 3, response rate: 88% of pharmacy students and 70% of medical students). The majority of students responded positively to statements on shared learning and interdisciplinary working, and there was a statistically significant difference in responses to three questions between cohorts (see Table 3, *p < 0.05,* two-tailed t-test).

### Thematic analysis of perceived learning

226/352 students returned the workshop evaluation form (response rate: 66% of pharmacy students, 62% of medical students). Student perceived learning gains from the workshops were assessed by the question ‘*Please state three things you learned from the session*’. Across all workshops, three major themes emerged from the analysis. These were:

- Knowledge acquisition
- Practical skills
- Reflection and deeper learning

**Fig. 1:**
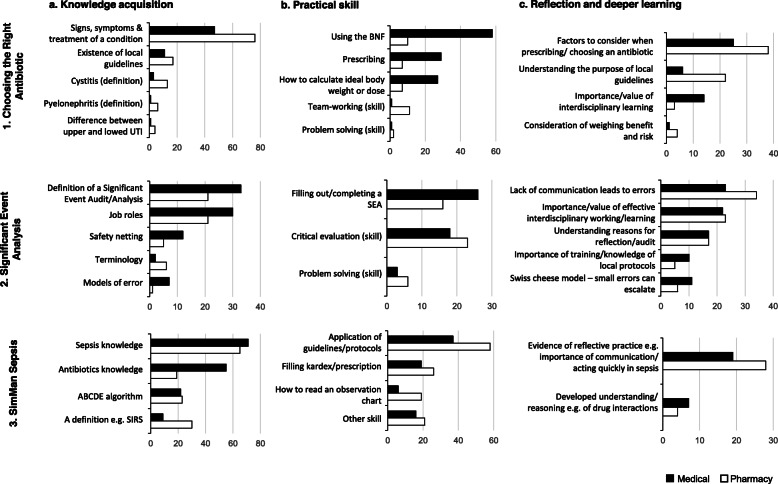
Thematic analysis of percieved learning gains from the three workshops. Charts illustrating the codes which make up a the three themes a: knowledge acquisition; b: practical skill; c: reflection and deeper learning, arising from student responses to the question *‘Please state three things you learned from the session’*, for each interprofessional workshop. The y-axes note the codes within a given theme for each workshop. The number of responses within that code are noted along the x-axes for medical (filled) and pharmacy (unfilled) students. Abbreviations: UTI, urinary tract infections; BNF, British National Formulary; SEA, Significant Event Audit; SIRS, Systemic Inflammatory Response Syndrome

Each theme was constructed from a number of related codes; Fig. 1 details the codes that make up a given theme. Below, we describe and compare the themes across the different workshops, presenting data extracts to illustrate the nature of the themes.

#### Knowledge acquisition

Students reported that they acquired clinical knowledge spanning the diagnosis and management of the conditions encountered, knowledge of the methods of appropriate antimicrobial prescribing, knowledge of job roles and models of human error. There were some profession-specific differences in students’ perceived *Knowledge* gains (see Fig. 1, panel 1a. 116 pharmacy versus 63 medical student comments). Free text comments illustrate this with pharmacy students stating they learned: ‘*Medical terms used for symptoms*’; ‘*What pyelonephritis is*’; ‘*Typical signs + symptoms of meningitis*’. Medical students, in contrast, more frequently reported knowledge acquired around the use of antimicrobials: ‘*I learnt the differences between empirical and second line antibiotics*’; ‘*Treat people close to a meningitis patient with prophylaxis*’.

Following the Significant Event Analysis (SEA) workshop students highlighted that they had learned what a significant event analysis/audit was, the concept of safety netting, models of error and about the roles of healthcare professions (see Fig. 1, panel 2a). These are exemplified in the medical students’ comments that they learnt, ‘*what safety netting actually encompasses*’ and ‘*how the Swiss cheese model works*’ and pharmacy students’ comments that they learnt ‘*what a Significant Event Audit is*’ and the ‘*different roles of healthcare professionals*’.

The major perceived learning in the SimMan sepsis session was reported to be knowledge around the identification and management of sepsis (including antimicrobial prescribing) and the ABCDE approach to managing an acutely unwell patient (see Fig. 1, panel 3a). Pharmacy student responses included that they learnt ‘*What sepsis is and how to screen for it*’ and the ‘*ABCDE method when examining a patient*’; medical students commented that they learnt ‘*necessary information for choosing an antibiotic for sepsis*’ and the ‘*assessment of acutely unwell patient*’.

#### Practical skills

Students reported that they learnt practical skills, most commonly around prescribing, such as how to write prescriptions in primary and secondary care, and the use of the British National Formulary (BNF) and local guidelines (see Supplementary File 1). The Choosing the Right Antibiotic (CRA) workshop showed a marked difference between cohorts in the reports of *Practical skills* learned (see Fig. 1, panel 1b, 116 medical versus 37 pharmacy student comments). Medical students stated that they had learnt ‘*how to look up suitable antibiotic treatments in the BNF’*, ‘*how to fill in prescriptions*’, and ‘*how to calculate the dosage for gentamycin for an obese patient*’.

In the SEA workshop, students reported that they had gained skills around how to conduct a significant event analysis, critical evaluation and problem-solving skills (see Fig. 1, panel 2b). Medical students reported ‘*learning skills to analyse situations*’ and ‘*how to identify where mistakes are being made, how to constructively identify possible improvements*’, while pharmacy students reported learning ‘*how to evaluate clinical situations*’ and ‘*how to identify a significant event*’.

Reported skills learned in the SimMan sepsis workshop included how to work with and apply local and national guidelines, how to fill in a hospital prescription chart (also known as, Kardex) and discharge prescription, and how to read an observation chart. These are exemplified in the free-text comments where pharmacy students stated they learnt ‘*how to fill in Kardex*’ and ‘*how to read a medical chart*’ and medical students reported learning ‘*the use of guidelines in the management of sepsis*’ and ‘*how to read a NEWS chart’*.

#### Reflection and deeper learning

Students reflected on their professional roles, identities and responsibilities after participating in all of the workshop sessions (see Fig. 1, panel c), but most frequently following the SEA workshop. Pharmacy students stated, for instance, that ‘*different professions had their limitations and stereotypes should not be made on their knowledge*’, that ‘*errors can happen but this can be minimised if there was teamwork between doctors, pharmacists and nurses*’, and the ‘*importance of not just working within your own profession/ use other professional to help*’. Medical students noted the ‘*importance of understanding other healthcare professional roles*’ and that ‘y*ou need to know everyone’s roles to work efficiently’.* Reflections on the importance of communication included ‘*importance of good communication in an IP team and how they rely on each other to provide the best care to patients*’ from a pharmacy student and ‘*communication between healthcare professionals is vital in patient care*’ from a medical student.

In the CRA workshop, students stated they developed an understanding of the factors to consider when choosing or prescribing antimicrobials (see Fig. 1, panel 1c). For pharmacy students, these included ‘*factors that can affect choosing the right antibiotic*’ and ‘*why regional guidelines vary from the BNF*’. For medical students, it included the ‘*importance of checking allergies and drug interactions before prescribing*’. Similarly, in the SimMan Sepsis workshops, students reported that they gained a greater understanding of drug allergies and interactions (see Fig. 1, panel 3c), exemplified by a medical student comment, that they learnt ‘*differentiating between a true and false allergy*’ and a pharmacy student comment that they started ‘*understanding the interactions that may occur as new medications are started*’.

### Thematic analysis of facilitators and barriers to learning, with suggestions for change

Facilitators and barriers to learning in each workshop, and suggestions for change, were assessed by the questions ‘*Please give examples of what you found interesting or helpful about this session*’ and ‘*Were there specific aspects of the session that hindered your learning? Please can you tell us what these were and what you think could be done to address these issues*’. Supplementary File 2 shows the themes that arose from the data analysis, and the number of responses within each theme by medical and pharmacy students. Below, we outline the major themes and illustrate these with quotes from the students’ free-text comments.

#### Learning and teaching techniques/resources

The workbook (Supplementary file 1), videos, SimMan and cases were frequently noted as helpful or interesting. This was evident in the medical students’ comments ‘*the case studies helped put everything into context*’, *‘the videos of each person involved helped to understand each person’s role and justification of the actions*’. Correspondingly, pharmacy students noted ‘*the case was interesting. It was a good idea to have one case running this session* [SEA] *and the SimMan case - better continuity*’, and ‘*I found the SimMan case most interesting. Seeing in real life what would happen in an emergency*’, and another comment that ‘*the options given to us when analysing the patient’s situation and understanding which ones were correct/ wrong and why, that was helpful*’ (see Supplementary File 1, p25).

Some pharmacy students commented that they did not know enough about the medical terms or diseases before the CRA session: ‘*We have not covered meningitis in uni yet, were in the dark about it*’. Pharmacy students also reported not having enough pre-knowledge of infections and sepsis before the SimMan workshop, and called for more use of SimMan in this session: ‘*Didn’t know what sepsis was, or how to diagnose it/ signs to look for so I felt rather useless in this session*’; ‘*Sessions were tailored around medical knowledge more than pharmacist knowledge*’ and ‘*The SimMan could have been used more to show examples of healthcare professionals - patient interaction*’.

#### Teamwork and reflection

Teamwork, including the skill of working with students from another discipline was noted as interesting and helpful. Medical students stated learning was facilitated by ‘*good teamwork with pharmacists*’ and that it was useful they ‘*taught each other in areas we’re not confident or experienced in*’, while a pharmacy student noted it was ‘*good working with med students who could diagnose the issue, whereas we could work through BNF to assign antibiotic*’. A few medical and pharmacy students highlighted a lack of engagement in the sessions by the other profession as a negative, as is evident in these comments: ‘*Only two medical students available in the session* [CRA] *therefore not enough input/ help from medic part*’ and ‘*Medical students display lack of interest and contribution*’. A medical student comment illustrates some of the difficulties working together: ‘*As before, I found pharmacists seemed reluctant to put their views forward/ were happy for medics to do a lot of the work*’.

Developing an understanding of the roles of the other healthcare professionals, and an understanding of how to learn from error, was another major theme that students found helpful or interesting. Pharmacy students appreciated ‘*how errors could be avoided with a better level of communication between members of the teams + patients*’, and ‘*understanding the different ways in which the patient could have received effective treatment*’. Some comments from medical students illustrate the value for them from these sessions: ‘*I thought it was really useful when we put ourselves in the positions of other health professionals and looking at mistakes from their viewpoint. It helped us appreciate sometimes mistakes do happen from other people and should be rectified as a team as soon as possible*’ and ‘*Analysing each individual’s role allowed me to see just how many people are involved in one patient’s case*’.

#### Logistics

Logistics was the biggest theme arising for barriers to learning, and suggestions for change, with comments that the group size was too big/rooms to small and time to short. An example of this is the comment from a pharmacy student, ‘*Lack of time to go through case studies more thoroughly*’. A medical student provided the following summary for the SimMan workshop: ‘*A bit crowded/ seating set up- got sat behind a pillar in the DR and struggled to see some things. Not space to move anywhere. Could be improved by either using different room or smaller groups*’. Other suggestions for improvement included having more medical students in each group, as some had low numbers.

## Discussion

This paper outlines the response of pre-clinical medical and pharmacy students to their first formal interprofessional education. We assessed the students’ perception of the value of IPE, learning gains and attitudes toward collaboration. We designed the conference using principles of contact theory, and employed pedagogical approaches that required balanced, collaborative input from both medical and pharmacy students. Students reported that the conference helped them to develop profession-specific knowledge and skills relevant to their future roles; in particular, medical students highlighted the acquisition of skills-based prescribing competencies while the pharmacy students highlighted that they learned about diseases and the diagnostic reasoning process. There was evidence that students had gained from the opportunity for interprofessional socialisation, learning about the roles and expertise of other healthcare professions [38], and they reported that the conference had demonstrated to them the importance of effective team work, collaboration and communication in ensuring patient safety.

### Theoretical frameworks

We found contact theory to be an effective framework for the development of our IPE activities [32]. Many of the positive findings from our evaluation came from consideration of the four core conditions proposed for effective interprofessional learning (see Table 1). Another common framework used in the analysis of IPE events is Biggs’ 3P (presage, process, product) model of learning and teaching [39]. Biggs’ original model has been developed more recently for analysis of IPE [40, 41]. This systems model approach explores the contextual factors that facilitate and hinder effective IPE. *Presage* factors include the learning and teaching context (e.g., institutional support and resource allocation), and teacher and learner characteristics including prior learning and beliefs. *Process* factors include the approach to learning and teaching, such as selection of teaching methodologies and facilitation style. *Product* is the outcome of the IPE initiative; the knowledge and skills acquired, or modification of attitude or behaviour. Principles of contact theory map onto the various components of the 3P model. We discuss our findings below within the context of these theoretical frameworks.

#### 3P framework and contact theory

The contact theory condition of institutional support for the programme and activities (Table 1, condition 4) is part of *presage*. In a previous evaluation of the logistics and feasibility of the IPE conference, developed in advance of this study for timely feedback to stakeholders, we noted local institutional support as being vital to the success of the event [27]. Regional and national support for the IPE conference, through involvement of professional bodies and senior NHS staff strengthened this support from authorities beyond the institutional level. Reeves and colleagues applied the 3P framework to an IPE project for community mental health teams. They identified *presage* factors, including lack of institutional support, as a key problem hindering the roll out of their pilot IPE initiative [42].

We considered equal status between groups in matching the year groups of the students and selection of LOs (see Table 1, condition 1). Learner characteristics are *presage* elements. Our approach to selection of LOs was constructivist, based on the belief that learners build new knowledge based on the foundation of what they have previously learned [43]. Thus, we mapped outcomes onto the students’ stage of development and current knowledge. The approach to interprofessional learning developed was both explicit and implicit, with LOs focussed on the clinical subject matter and on IPE. The creation of explicit and implicit curriculum content was a key recommendation of Shrader and colleagues in their Interprofessional Education and Practice Guide [34]. Learner expectations as well as learner knowledge are *presage* factors that can contribute to IPE success [42]. Students generally enter IPE programmes with positive expectations, with younger students reported to be more positive in their interprofessional attitudes [44]. This was our students’ first IPE experience, and our results support previous findings of initial positive attitudes towards IPE (see Table 3) [41, 45]. The majority of students felt ready to engage at this pre-clinical phase of their development. Previous research has highlighted the potential benefits of early exposure to IPE [46, 47].

Students were keen to see an increase in IPE events in the curriculum (see Table 3). The conference format was developed for our pilot early-years IPE initiative, in part, as a mode of bringing together large and geographically separated cohorts of students [27]. However, in line with this student feedback, the evidence ‘*supports the needs for multiple exposures to maximise sustained learning and change*’ [33]. We need to develop frequent, smaller scale events that run longitudinally through the curriculum. In this study, we did not map our outcomes against an IPE competency framework [48]. However, as our curricula develop, it will be important to do so to ensure all IPE competencies are adequately addressed [34].

In many IPE events, student participation is voluntary, which may bias towards participants already more open or amenable to IPE [41, 49, 50]. Our event was compulsory, as all small group sessions in our programmes are, which should reduce this bias, although not all students chose to participate in the evaluation. We aimed to achieve equal numbers of students in each workshop. However, around 15% of medical students did not attend on the day, unbalancing numbers. Absences were presumed to be due to the conference being on the last day of term, and the day after the hand in of the last piece of in-course assessment for the year. This highlights the importance of considering all *presage* factors such as the timing of events in the development stage. Low numbers of medical students in some small group sessions, and a lack of engagement from others who were present was noted in the evaluation as an area for improvement. This, together with a feeling from some pharmacy students that they did not have sufficient medical knowledge before the conference, may in part explain why pharmacy students responded less positively to three of the statements evaluating the value of the interprofessional nature of conference (see Table 3).

IPE can reinforce negative stereotypes if these are enacted in the sessions [51], and negative attitudes towards medical students from non-medical healthcare students can remain unchanged or be reinforced following IPE exposure [49]. Overall, our data indicate that the conference did help the students to understand the value and expertise of other professionals. However, since our study did indicate some negative experiences due to lack of engagement by a minority of students, we propose explicit teaching in advance on professional identity formation and the potential for IPE to reinforce or ameliorate negative stereotypes. This could be in the form of an online IPE tutorial that is embedded within the curricula and a prerequisite to attendance at the first shared IPE event.

Workshop tasks were designed for students to work collaboratively not competitively, on common goals (see Table 1, conditions 2–3). This co-operative approach to learning is a *process* factor [41]. The two cohorts came to the conference with different levels of skills and knowledge; pharmacy students with knowledge of prescribing and the medical students with basic knowledge of antimicrobials. The CRA session in particular was specifically designed to require balanced but differing input from the two cohorts, such that the tasks were beyond the capabilities of an individual cohort but achievable when subject-specific skills and knowledge from each profession were combined. The success of this approach is illustrated in Fig. 1, where medical students more frequently stated they learned practical skills from the pharmacy students (e.g., how to use the BNF) and the pharmacy students more frequently stated they had gained knowledge from the medical students around the diagnosis and management of infections.

The use of simulation, applied clinical cases, video-based learning and facilitation are all *process* factors that evaluated positively in our study. There is evidence that positive outcomes for IPE depend on students regarding activities as authentic experiences which replicate the clinical workplace and interactions [51]. The SimMan Sepsis workshop was a combination of simulation, role play and interactive team voting activities; these were designed to elicit maximum engagement with the scenario and imitate the pressures and complexities of managing an acutely unwell patient [26]. A review of simulation-based IPE found that most studies revealed positive outcomes related to student satisfaction and their perceptions of learning, which our study supports [24]. There is evidence that simulation is superior to traditional clinical medical education in achieving specific clinical skill acquisition goals [22]. However, most studies, including ours, did not assess knowledge and skills pre- and post-intervention, nor compare these to a control group, so were unable to determine whether the perceived learning gains were achieved and were related specifically to the simulation intervention [21, 24].

#### 3P framework and Kirkpatrick’s educational outcomes model

The outcomes of our IPE event, the *product*, map to levels 1 and 2 of Kirkpatrick’s educational outcomes model [41, 52]. We evaluated the participants’ reaction to the workshops, assessing their views on the content, teaching methods and organisation (Kirkpatrick Level 1). Analysis of the specific aspects of the workshops that most helped or hindered their learning is informing future development of the sessions. Specifically, we have built in more elements explicitly exploring interprofessional teamwork, and have simplified some of the clinical elements of the scenarios. We assessed students’ attitudes toward the value of interprofessional collaboration in education and patient care (Kirkpatrick Level 2a) [41]. Development of an understanding of other healthcare profession roles, the importance of interprofessional communication and effective interprofessional collaboration to ensure patient safety were major themes that arose from our evaluation. We argue that this could not have been achieved as effectively in a uni-professional intervention or through solely didactic teaching.

We assessed students’ perceived acquisition of knowledge and skills (Kirkpatrick Level 2b). Students reported acquisition of problem solving and critical evaluation skills, and a wide range of knowledge including concepts and procedures related to infection management and antimicrobial prescribing. When teaching prudent antimicrobial prescribing, educators are advised to adopt a competency-based approach that develops practitioners who are knowledgeable, skilful and reflective [53, 54]. Davenport and colleagues suggest that outcomes include the ability to carry out practical procedures, undertake patient investigations, handle and communicate information and facilitate the development of decision-making and clinical reasoning skills [54]. These were all themes that emerged from our student evaluation, suggesting that our simulation and interprofessional workshops are appropriate approaches for teaching antimicrobial prescribing.

### Strengths

The major strength of the study was in the design of the interprofessional intervention. It incorporated positive contact principles and many of the features that underpin effective IPE activities as outlined by Teodorczuk and colleagues in their Toolbox article [19]. These include oversight of the intervention by an interprofessional collaborative steering group, development of common learning items, focussing on authentic learning activities, training of the facilitators and coupling simulation with IPE. In addition, learning was comprehensively evaluated across both cognitive psychomotor and affective domains. A further strength of the evaluation was the use of the 3P framework which enabled a systematic exploration of factors that facilitate and hinder IPE activities.

### Limitations

A limitation of our evaluation is the self-perceived nature of learning and changes in attitude; we did not conduct a pre- and post-test to evidence the learning that took place. We also did not use a control group of students covering the same material in a non-simulation and non-IPE context, thus, we cannot conclude that these approaches are more effective that other traditional or uni-professional teaching approaches [24]. However, for an event of this magnitude, it was unlikely that students would be accepting of being randomised to a control group for fear of missing out and issues of equity would likely arise. We did not use an established validated measure such as the Readiness for Interprofessional Learning Scale (RIPLS) to assess interprofessional learning because this study already included a large-scale evaluation of the conference format and workshops, so we developed a shorter quantitative survey on the value of IPE. This survey was informed by the RIPLS and had been developed iteratively over previous IPE evaluations at our institutions. The evaluation only assessed students’ attitudes at the time of the conference. A follow up evaluation, after they had entered the clinical years, would have enabled us to assess any impact of the conference on changes in attitude and behaviour in practice, but was beyond the scope of this study [41, 55].

Pharmacy students felt they lacked sufficient clinical knowledge before some of the workshops. Although we had designed the workshops to draw on the different strengths of the cohorts, we did not brief the student about this sufficiently. We need better signposting of professional roles in the session or more previous teaching of the clinical concepts for pharmacy students. Early IPE events before the conference, on explicit IPE themes such as teamwork or professional roles, should help integration of students at the conference.

### Future work

The conference and workshops have been developed in format and content in subsequent years, due to: results from this evaluation, a programme merger and curriculum renewal in the medical programme, and addition of a pharmacy programme at Newcastle University. As indicated above our study afforded learning at Kirkpatrick levels 1 and 2. The next step is to demonstrate learning at Kirkpatrick level 3. However, achieving such learning at scale is traditionally challenging. Moreover, our learners were at prequalification level. Such an approach will need to be carefully designed to achieve demonstrable, reliable and valid results in a randomised controlled trial or other experimental setting. Arguably the conference would need to be repeated to sustain learning, embedded within an IPE framework of both ‘CAIPE compliant’ [19] and non-compliant learning activities that progressively builds collaborative capabilities and underpinned with sound educational theory as described previously. Such an approach could lead to IPE success that later demonstrates changes to prescribing behaviour and potentially health benefit.

## Conclusions

The conference was an innovative response to the challenges of delivering healthcare profession education around infection management and antimicrobial prescribing. Employment of interprofessional and simulation pedagogies allowed exploration of the contextual factors that affect the safe and effective management of infections. Using contact theory and the 3P framework in the design and analysis of our study enabled a more complete understanding of the barriers and facilitators to learning. Students reported positive changes in knowledge, skills and attitude towards interprofessional working and antimicrobial prescribing that aligned with positive contact principles. Our description and analysis of the workshops can facilitate others in the development and validation of novel simulation and interprofessional teaching activities for antimicrobial education.

## Supplementary information

**Additional file 1.** Interprofessional Education Conference Workbook. The Interprofessional Education Conference Workbook, from the 2016 pilot conference evaluated in this study. The cases within the workbooks were given as pre-reading to the students. On registration students were provided with this full workbook, which contains materials and tasks for the day, including blank prescription forms and other relevant documentation.

**Additional file 2.** Facilitators and barriers to learning in each workshop. Thematic analysis of student free text responses to questions about facilitators and barriers to learning in each workshop. The table shows broad themes that arose from the data analysis, details about what each theme encompasses, and the number of responses within each theme by medical and pharmacy students. Abbreviations: MCQs, Multiple Choice Questions.

## Data Availability

The datasets used and/or analysed during the current study are available from the corresponding author on reasonable request.

## Abbreviations

IPE: Interprofessional Education
UG: Undergraduate
LOs: Learning Outcomes
BNF: British National Formulary
CRA: Choosing the Right Antibiotic
SEA: Significant Event Analysis
