# Supplementary material for: Designing and evaluating an interprofessional education conference approach to antimicrobial education
Source: BMC Med Educ. 2020 Oct 13;20:360. doi: 10.1186/s12909-020-02252-9 (PMC7552509; doi:10.1186/s12909-020-02252-9)
Supplement: Supplementary file 1 — Additional file 1. Interprofessional Education Conference Workbook. The Interprofessional Education Conference Workbook, from the 2016 pilot conference evaluated in this study. The cases within the workbooks were given as pre-reading to the students. On registration students were provided with this full workbook, which contains materials and tasks for the day, including blank prescription forms and other relevant documentation. [file 12909_2020_2252_MOESM1_ESM.pdf]

INTERPROFESSIONAL EDUCATION CONFERENCE 2016

Friday 11th March 2016

Contributors: Clare Guilding, Louise Statham, Elsa Randles, Jo Matthan, Alan Green, Jess Hardisty
Logo designed by Saad Khan and Will Sparkes

IPEconference 2016

© The University of Sunderland, Newcastle University

Interprofessional Education (IPE) conference: Introduction to IPE

What is IPE?

The Centre for Advancement of Interprofessional Education (CAIPE) http://caipe.org.uk/

defines IPE as:

> "when two or more professions learn with and from and about each other to improve collaboration and quality of care"

Why is IPE important?

Traditionally, healthcare professionals were trained completely separately within their different professional groups. The expectation was that once qualified, you would work together in multidisciplinary teams in what could be a very hierarchical structure. This model no longer works due to a number of factors:

- **Increasing complexity in healthcare** – no one professional group can expect to hold all the necessary expertise to manage patients with complex health and social needs.
- **Increasing crossover and complexity of roles** – with the European working time directive effectively reducing health professional availability and the increasing costs of healthcare causing NHS trusts to try to reduce the payroll burden, there has been an increasing need for broadening of roles within professions and blurring of traditional role boundaries between the health professions.
- **Rapid patient turnover/ increasing community care** – more patients are living longer with complex health needs, and hospital stays are shortening. This means that more patients are 'out there' living in the community with very complex needs.
- **Increasing accountability** and increasing patient expectations.

In order to address these changing needs and to ensure patient safety (reducing risks to both patients and professionals) it is imperative that we learn together. Part of this learning is around collaborating successfully, in order to provide effective care, improve patients' and families' perceptions of care and improve our working lives.

What are the aims of IPE in undergraduate training?

At this early stage in your training, the aim is to prevent the development of a 'silo mentality' by providing opportunities for you to work collaboratively with students from other healthcare professions. This should make it easier for you to adapt to and enjoy multidisciplinary teamwork during your clinical training and in your future career. This conference should enable you to:

- Improve your understanding of professional roles and boundaries.
- Understand and value the expertise and values of other team members.
- Enhance your communication skills with other professionals.
- Experience working in a multidisciplinary team to solve clinical problems.

Links to resources around IPE

- WHO Patient Safety curriculum guide (multiprofessional) available at: http://whqlibdoc.who.int/publications/2011/9789241501958_eng.pdf?ua=1
- The Mid Staffordshire NHS Foundation Trust Public Inquiry (Francis, 2010) available at: http://www.midstaffspublicinquiry.com/
- Laming Report (2009) available at: http://webarchive.nationalarchives.gov.uk/20130401151715/https://www.education.gov.uk/publications/standard/publicationdetail/page1/HC%20330
- Centre for Advancement of Interprofessional Education (CAIPE) available at: http://caipe.org.uk/
- Introducing Interprofessional Education (2013) available at: http://caipe.org.uk/news/caipe-announces-its-latest-publication--introducing-interprofessional-education/
- Interprofessional learning: A pocket guide for mentors, facilitators and educators of all professions available at: http://caipe.org.uk/news/interprofessional-learning-a-pocket-guide-for-mentors-facilitators-and-educators-of-all-professions/

Outline of the day

Timetable

You each have an individual timetable which has been emailed to you and is contained in your conference bags. Please ensure you attend only the sessions which are in your timetable.

Time	Session	Venue
8:00-9:00	Registration	RB Green Lecture Theatre foyer
9:00-10:00	Introduction and keynote speakers	RB Green Lecture Theatre
10:00-12:00	Individual sessions	See individual timetable
12:00-13:30	Lunch and society stalls	See individual timetable
13:30-15:30	Individual sessions	See individual timetable
15:30-16:15	Round up and evaluation	RB Green Lecture Theatre

Keynote speakers

Our keynote speakers both work regionally within the NHS and will speak about the value of good interprofessional working practice, how this can impact on the outcome for a patient and their experiences of interprofessional working in the NHS. Our speakers are:

– **Chris Tiplady**, Regional Advisor for Education - Health Education England North East
– **Neil Gammack**, Chief Pharmacist, Gateshead Health NHS Foundation Trust

Individual sessions

There are 3 individual sessions you will complete on the day:

1.	Choosing the right antibiotic	p6-18	Ridley Building 2
2.	Significant event anaysis	p19-24	Leech Building
3.	SimMan sepsis	p25-43	Clinical Skills or Dissecting Room

Look at your timetable to find out the specific time and room you are doing each session in.

Lunch

Lunch will be held from 12:00-13:30. Please only collect lunch from the venue on your individual timetable. Lunch can be eaten in the undergraduate common room, the Baddiley-Clark seminar room, the Ridley seminar rooms or the Forum. In the Forum customers buying food there take precedence for the seating. If eating in teaching rooms PLEASE throw your rubbish away.

Stalls

We have six organisations who are running stalls on the day. Please use the lunch period to visit these stalls. Many of them have information about student memberships, some of which are free and enable you to apply for travel bursaries for conferences.

CAIPE (Centre For The Advancement Of Interprofessional Education Accessibility)

CAIPE is an independent 'think tank' comprising of individual, corporate and student members working with organisations in the UK and overseas to improve collaborative healthcare practice and thereby the quality of patient care by professions learning and working together. CAIPE are developing and pioneering a student network for those engaged in or researching Interprofessional Education or interprofessional collaborative practice. The network aims to support, advise, and nurture all aspects of Interprofessional Education.

UKCPA (United Kingdom Clinical Pharmacy Association)

The UK Clinical Pharmacy Association (UKCPA) is a member association for clinical pharmacy practitioners. They encourage, support and promote advanced practice in pharmacy. The UKCPA actively develops clinical pharmacy practice as well as developing individual practitioners, and are frequently at the forefront of initiatives such as establishing professional curricula, developing professional recognition (credentialing) processes, and developing professional tools and frameworks for practitioners.

BPS (British Pharmacological Society)

The BPS is a charity with a mission to promote and advance the whole spectrum of pharmacology. The BPS and MSC Assessment work together to deliver the Prescribing Safety Assessment (PSA) that all final year medical students must take, and that forms a summative part of the assessment for Newcastle University's Stage 5 MBBS students. The BPS campaigns to increase the awareness of clinical pharmacology, highlighting that it is uniquely placed to deliver the key strategic priorities for the NHS. These include: protecting patient safety; delivering person-centred care; leading medicines research; promoting access to innovative medicines; ensuring medicines are cost-effective and used to best effect; and training skilled and safe prescribers.

NPA (National Pharmacy Association)

The NPA is the trade association for pharmacy professionals in the UK. Their aim is to support independent community pharmacies to succeed professionally and commercially for the benefit of their patients. As a not-for-profit organisation they are committed to reinvesting in service provision.

PDA (Pharmacy Defence Association)

The PDA look after the interests of individual pharmacists. They defend pharmacists when faced with a conflict, and proactively lobby the individual pharmacist's agenda. The PDA is the only organisation solely looking after the interests of individual community, primary care and locum pharmacists in an increasingly difficult and hostile environment. The PDAs aims and objectives are to support and protect pharmacists' reputation as they practice their profession and to improve the status and working environment of individual pharmacists to improve patient care.

MPS (Medical Protection Society)

MPS is the world's leading defence organisation for doctors and healthcare professionals. More than 90% of medical students in the UK are members, and they protect and support more than 300,000 members in the UK and beyond. They are much more than a last line of defence and believe prevention is better than cure. They provide advice and support to help avoid risks in the first place. The MPS are the kind sponsors of our IPE conference logo competition.

Learning outcomes

The conference will cover the following outcomes from the MB BS and Pharmacy programmes:

Sunderland

MPH209/210

- Apply the acquired integrated knowledge and skills to patient-focused pharmaceutical care, with due regard to critical awareness of clinical literature, current evidence-based management guidelines and standard operating procedures

MPH210

- Understand the safe and effective supply of medicines and appropriate related products in primary and secondary care including concepts around clinical governance including audit, risk, patient safety, error reporting and the context of CPD within a professional governance framework

MPharm

- Essential knowledge of pharmacy-related aspects of fundamental pharmaceutical and human sciences and how this material relates to patient care
- Essential knowledge of the normal anatomy, physiology and function of the human body including an understanding of associated nomenclature and notation
- Critically evaluate the prescribing decisions of others
- Understanding of the context for pharmaceutical intervention within the wider sphere of patient care and multidisciplinary health teams; it's practice and problems

Newcastle

CPTP3

- Describe the rationale behind the selection of an appropriate antibiotic in meningitis
- Describe the principles of antibiotic use
- Explain the importance of collaboration with pharmacists in preventing errors

TSM

- Describe the clinical signs and symptoms of meningitis

CSIM1

- Describe the symptoms and signs of urinary tract infections (UTI)
- Describe the antimicrobial treatment of urinary tract infections
- Define the following terms: - bacteraemia, systemic inflammatory response syndrome (SIRS), sepsis and septic shock

PDS4

- Identify the professional roles of members of the healthcare team in hospital and community settings
- Define medical error and describe the link to patient safety
- Describe ways to respond to medication error as a doctor
- Explain how prescribers can reduce prescribing error

Choosing the right antibiotic

Session aim

To enable students from different health professions to work together, utilising their combined knowledge to solve clinical problems around the selection and administration of antibiotics.

Session outline

This is a 2 hour seminar in which groups of medical students and pharmacy students will work together to solve clinical case-based prescribing problems. Each group will be co-facilitated by a medical doctor and a pharmacist, and will consist of ~18 students (with an even mix of medical and pharmacy students) broken down into mixed sub-groups.

Real prescription forms for both outpatient and inpatient prescriptions and clinical guidelines will be provided for each group during the session. An answer sheet will be made available after the session, so please concentrate on completing the tasks in your groups and don't worry too much about making notes.

The following is additional information, which you have been given as pre-reading, about the clinical cases you will be working on during this session.

A woman with painful micturition

Presentation

Joan Richardson was a 54 year old woman with a history of recurrent urinary tract infections. On the day in question, she noticed symptoms of burning pain when micturating, and that her urine was cloudier and smelling more strongly than usual. In addition she began to experience urinary frequency, and spent the rest of the morning 'going little and often'.

She recognized these symptoms as being typical of cystitis, and made an urgent appointment with her General Practitioner, who was able to see her that afternoon because of a cancellation. In the meantime she began to address her symptoms as she always did, by drinking more fluids.

Her GP examined her and found mild suprapubic tenderness, and evidence of proteinuria and haematuria on dipstick testing of her urine. Mrs. Richardson provided a mid-stream specimen of urine, taking care to avoid contamination from the skin, and this was submitted to the laboratory the same afternoon by the regular 4 o'clock courier. The GP felt that Mrs. Richardson's symptoms were sufficiently severe to warrant empirical antimicrobial therapy. Mrs. Richardson went straight from the surgery to the chemist's and picked up the antibiotics.

The next day she woke feeling less well, with an aching pain that seemed to arise in her left flank. She felt cold and shivery, and was not surprised that her temperature was 38.5° C. Her dysuria persisted, and her urine remained cloudy. She wasn't sure whether to persist with the antibiotics, which after all she had only started the evening before, or whether to seek a further opinion from her GP. She decided to continue with the antibiotics, and to return to bed.

She was telephoned early that afternoon by the practice nurse from the surgery, who informed her that the microbiology report on her urine specimen had come through on the LabLink electronic reporting system. Microscopy had shown over 200 white blood cells per ml of urine, and culture had shown over 100,000 coliform organisms per ml of urine. Antibiotic sensitivity testing had been performed, and the isolate was resistant to cephalexin and trimethoprim but sensitive to nitrofurantoin, co-amoxiclav and ciprofloxacin.

Mrs. Richardson explained that she was no better, and in fact felt rather worse, and the practice nurse offered to make her an emergency appointment at the evening surgery. Her husband drove her up, and the GP confirmed her fever, and found marked tenderness in the left renal angle. A diagnosis of pyelonephritis was made, and the GP discussed with Mrs. Richardson whether or not she should be admitted to hospital. It was decided to treat her at home with a different antibiotic to which the organism was sensitive, but to keep a close eye on her.

Over the next two days Mrs. Richardson appeared to improve initially, but she remained intermittently feverish, nauseous and became very alarmed when she began to have attacks of violent shaking. Her GP visited her at home, and was sufficiently concerned to admit her to the surgical department of the local hospital.

Examination and investigations:

On admission she was febrile (38.0° C) and flushed, with exquisite tenderness in the left renal angle but no evidence of peritonism. Examination was otherwise unremarkable.

A full blood count showed a neutrophil leucocytosis consistent with an acute bacterial infection. A biochemical profile showed a raised urea consistent with a degree of dehydration, but a normal creatinine. C-reactive protein, an acute phase protein used as an inflammatory marker, was markedly elevated. Further urine cultures were taken, as was a blood culture. Abdominal ultrasound was organised for the next day.

Treatment:

The diagnosis made on admission was acute pyelonephritis, with the possibility of perinephric abscess. Antibiotic advice was taken from a microbiologist, who recommended starting ciprofloxacin intravenously pending the culture and ultrasound results, and giving her a single dose of intravenous gentamicin 5mg/kg.

The next day her urine was reported as still showing over 200 white blood cells per ml, but culture was negative. However, her ultrasound findings were consistent with pyelonephritis and an associated perinephric collection. The blood cultures remained negative.

The abscess was drained by insertion under ultrasound guidance of a percutaneous ‘pigtail’ catheter. 50mls of pus emerged, and from this was subsequently cultured a pure growth of *Escherichia coli*, resistant to amoxicillin but sensitive to ciprofloxacin and gentamicin. Almost from the point at which the catheter was inserted, Mrs. Richardson began to feel better than she had for days. Her fever settled over the next 24 hours, and she was changed from IV to oral ciprofloxacin in anticipation of her discharge home from hospital.

A teenager with a headache

Presentation

Sophie Masters was a 19-year-old student in her second year at Newcastle University where she was studying geography. She lived in a flat in Jesmond with three other students one of whom was a second year medical student. Sophie woke up one Monday morning in term time not feeling very well. She got up in good time for her 10 o'clock lecture but felt weak, had aches in her muscles and back and was feverish. She decided that she had probably got 'flu and went back to bed. When her flatmates came back that evening Sophie was still in bed. Since she was really quite poorly and was usually a pretty fit person, they persuaded her to call her GP. Dr Asher agreed, somewhat reluctantly, to visit after evening surgery. She finally arrived at 7pm - it had been another 'surgery from hell' and she was feeling tired and not a little stressed - and rather curtly took Sophie's history. She carried out a swift but thorough examination, said she thought it was indeed 'flu, and that 'there was a lot of it about'. Sophie should continue to take paracetamol for fever and pains, and she should be better in a couple of days.

The next morning Sophie woke after her flatmates had gone in to university and when she tried to get up she had a headache which didn't seem to come from one specific part of her head but felt as if it was all over. She went back to bed and over the next couple of hours the headache became much more severe and got worse if she tried to move about. She telephoned her mum in Northwood, Middlesex for some sympathy and advice. Her mum told her to continue taking paracetamol and to try and sleep it off. However shortly after taking the painkillers she became nauseous and had to rush to be sick down the toilet. Over the day her headache became much worse and she could no longer tolerate light which made her eyes hurt. She closed the curtains in her room and tried to lie as quietly as she could.

When her medic flatmate Anna came home, she noticed that Sophie looked flushed and when she turned the lights on Sophie appeared to flinch and tried to hide her eyes beneath the blanket. She noticed that Sophie appeared to be a little confused and not really 'with it', but more worryingly had a few red spots on her legs and buttocks. Anna began to suspect that Sophie had more than just 'a touch of flu', and when she pressed an empty glass over the spots, to her horror, it did not go pale. She realised she needed emergency treatment and called a 999 ambulance to take her into the Accident and Emergency department at Newcastle General Hospital. Anna left a note for her flatmates and accompanied Sophie in the ambulance.

Examination

On arrival at A&E Sophie was examined first by the triage nurse and then by one of the casualty officers. She appeared flushed and looked ill. She had obvious photophobia, keeping her eyes closed or pulling the blanket over her head in bright light. Her temperature was 38.5˚C. By this time the rash had become more extensive over her legs, buttocks and trunk. On attempted flexion of her neck she displayed an involuntary muscle spasm causing her neck to be stiff. With Sophie lying on her back the doctor attempted to flex her hip and then extend her knee. This caused Sophie to develop exquisite pain in her back. Fundoscopy was normal. She had no focal neurological abnormalities (weakness, disturbance of sensation or coordination in her limbs). Her blood pressure initially on admission was 105/65 with a pulse rate of 100. Half an hour later her blood pressure dropped to 85/60, her pulse rate rose to 130 and her extremities became cool.

Diagnosis and Initial Treatment

Because of the short prodromal illness followed by increasing headache and signs of 'meningism' (photophobia and neck stiffness) a diagnosis of meningitis was reached. The evolution of the problem and its increasing severity suggested it was a bacterial meningitis. The commonest agent causing bacterial meningitis in a young adult is *Neisseria Meningitidis* and the haemorrhagic rash that had developed on her trunk was strong clinical evidence to support this. Sophie was becoming increasingly unwell. Her flatmate telephoned her parents to inform them. The development of hypotension, increasing pulse rate and a shut down in the peripheral circulation suggested that she had an associated septicaemia. This is a life threatening condition which required immediate admission to hospital, urgent treatment and investigation. Treatment was started in A&E: including intravenous fluids to counter the hypotension and immediate parenteral antibiotic treatment in high doses. Sophie was admitted from A&E to a medical ward and the following investigations were carried out.

Investigations

A full blood count showed a neutrophil leukocytosis indicative of an acute bacterial infection. A clotting screen was carried out as it is known that a proportion of patients with meningococcal septicaemia will develop the complication known as disseminated intravascular coagulation in which the patient's clotting system is deranged. The presence of a haemorrhagic rash over Sophie's limbs and torso raised this as a possibility. The results of this test were within the reference range. Blood samples were sent to the Microbiology Lab for blood cultures to be performed in order to try to isolate the infective organism.

Lumbar puncture was performed to extract a sample of cerebrospinal fluid (CSF). This was obtained by inserting a needle between the spines of the lumbar vertebra into the thecal sac. The CSF when withdrawn appeared turbid rather than the clear colourless acellular fluid that is normally found. This was further indication of a bacterial reaction within the meninges. Microscopy showed a cell count of more than 1000 polymorph leukocytes per cubic millimetre of CSF. Biochemical analysis of the CSF revealed elevated protein content and reduced glucose content.

U&E screens (urea and electrolytes) were performed at regular intervals to make sure that electrolyte balance was maintained and that renal function was not becoming impaired by the hypotension. Direct microscopy of a smear of centrifuged CSF stained with Gram's stain revealed the presence of Gram negative diplococci suggestive of the presence of Neisseria Meningitidis. Within 24 hours a culture of the organism isolated from the CSF sample taken from Sophie was obtained and it was shown to be sensitive to a number of antibiotics.

Whilst upset by the serious nature of her friends problem, Ann had been very impressed by what she had seen in A&E – much more interesting than sitting in a lecture theatre!

Treatment

Sophie's antibiotic treatment was modified to provide a cocktail of antibiotics to which the bacteria were known to be sensitive and she was maintained on intravenous fluids. After two days of treatment Sophie began to feel better and her headache had almost disappeared. The rash had begun to disperse and her blood pressure had returned to 125/85. After a week in hospital she was well enough to go home where she was confined to her sick-bed for a further couple of weeks, but being a conscientious student resumed her studies by getting her friends to bring work home for her.

Public Health

The hospital clinician, having identified meningitis, was required by law to notify the proper officer of the local authority, who is usually the Consultant in Communicable Disease Control (CCDC). The day after admission, the CCDC arranged to interview Sophie to identify all those who had been in close contact. All of Sophie's flatmates, her boyfriend and those who had stayed overnight in her flat were given chemoprophylaxis to eradicate the infecting strain of *Neisseria Meningitidis* from the network of contacts, and thus prevent further cases among susceptible close contacts. In addition, the CCDC notified the university advising them to circulate notices to all students in Sophie's year to be on the alert for possible symptoms of meningitis. No further action was recommended at this stage following a single case.

A 'near miss'

Sophie's parents were informed about her illness while she was incapacitated as this was considered to be in her best interests. They had got over the initial shock of finding out about her illness and dropping everything to speed north to her hospital bedside; they pieced together the course of events. They were very distressed and angry to hear about the seemingly cursory visit from the GP, who had diagnosed 'flu, and missed a case of potentially fatal meningitis - in *their* daughter. Although the consultant explained to them that it was a notoriously difficult diagnosis to make in the early stages of the disease, which reassured them a little, they still wanted to find out what had happened, and if necessary make a complaint about the GP's care. They went to the surgery and asked if they could see the doctor to get an explanation. They were told that she was not in surgery that day, but were seen straight away by the practice manager. She had a brief chat with them, outlined the procedure if they wished to make a formal complaint – which they said they didn't at this stage – and she promised to arrange a meeting as soon as possible.

Meanwhile, a couple of days earlier, the casualty slip documenting Sophie's admission arrived in the practice mail. When she read it Dr Asher was very upset, realising she had missed a diagnosis of meningococcal meningitis/septicaemia, which could have dreadful consequences for the patient. She remembered how she had been rushing to get home after a gruelling day and wondered whether she had missed some glaring physical signs, such as the classic purpuric rash. The fact that it was clear, from a quick phone call to the ward, that Sophie had been quite lucky in being diagnosed and treated 'just in time' did not make Dr Asher feel any easier about it. She talked things over (and over and over!) with her husband, also a GP, and with her partners, and began to resolve some of the issues.

By the time she met with Sophie's parents a few days later, in the company of the practice manager, all parties had begun to see things more rationally. Sophie's parents were struck by how genuinely concerned and distressed by the incident Dr Asher was. They accepted her explanation of events and her apologies, but also realised that *no* doctor would probably have diagnosed meningitis at the point she visited Sophie that evening. Although exonerated of blame in terms of her technical care, Dr Asher reflected on the fact that a doctor must be vigilant at all times, no matter how tired and stressed they feel. She also reflected on the fact that a doctor's manner often affects patients profoundly.

She remained upset by the incident for some time, recognising herself to be somewhat over-vigilant in her management of similar situations. She eventually contacted a local support organisation for GPs called Primary Care Choices, and had a couple of sessions with a mentor, which helped enormously, in particular in helping her come to terms with the fact that errors and mistakes (and 'near misses'!) are inevitable in medicine. All we can do is to strive to prevent them. Also, recognising that medical errors often have psychological and emotional consequences for the doctor, as well as the patient and

their family, using the Egan model, the mentor helped Dr Asher think through a strategy for handling such situations in the future using the Egan model.

- There are approximately 3,500 to 4,000 reported cases of meningitis per year in the UK.[1]
- Meningitis can be caused by bacterial, viral or fungal infection.[2] Viral infection is the most common cause but is usually less severe.[1] Enteroviruses cause 80-90% of diagnosed cases of viral meningitis.[3]
- In most countries, more than 50% of cases of spontaneous community-acquired meningitis in adults are caused by Neisseria meningitides and Streptococcus pneumoniae.[3]
- Listeria monocytogenes, aerobic Gram-negative bacilli (such as Escherichia coli), Haemophilus influenzae (Hib), and staphylococcus aureus cause most of the remaining cases.[3]

References

1. Department of Health. What is meningitis? 2006. Available from: http://webarchive.nationalarchives.gov.uk/+/www.dh.gov.uk/en/AboutUs/MinistersAndDepartmentLeaders/ChiefMedicalOfficer/ProgressOnPolicy/ProgressBrowsableDocument/DH_5852375
2. Kumar P, Clark M (editors). (2004) Clinical Medicine, London: Elsevier.
3. Warrell, D, Cox, T M, Firth, J D, Benz, E J. (2004) Oxford Textbook of Medicine, Oxford: Oxford University Press.

TASKS

A woman with painful micturition

<u>Pyelonephritis case</u>

Task 1:

<u>What are the medical terms for the symptoms Joan experienced?</u>

<u>What is the most likely diagnosis for this patient?</u>

<u>Dipstick test results</u>

Nitrites: The test for nitrites is a screening method for infections caused by nitrate-reducing bacteria. Some of the gram negative bacteria species that most commonly cause urinary tract infections (Escherichia coli, Enterobacter, Klebsiella, Citrobacter and Proteus) have enzymes that reduce the nitrate present in urine to nitrite

Protein: A positive test for protein can indicate the presence of bacteria – it can also be indicative of other diseases of the urinary tract or kidneys

Leukocytes: A positive test for leukocytes normally indicates the presence of bacteria

Haemoglobin: A positive test for haemoglobin can indicate trauma to the kidneys or urinary tract – it can also be as a result of infection

<u>What does ‘empirical’ mean?</u>

Task 2:

Write up the antibiotic prescription on the FP10 form provided

Pharmacy Stamp<br><br>Please don't stamp over age box	Age<br><br>D.O.B	Title Forename, Surname & Address
Number of day's treatment		
Endorsements		
Signature of Prescriber		Date
For Dispenser No. of Prescns.	WORTOON CENTRAL PRIMARY CARE TRUST<br>Dr H Ferriman<br>HEALTH CENTRE<br>COUNCIL AVENUE	
NHS	015520000012	

Task 3:

Why is Joan feeling worse despite the antibiotics?

What factors need to be considered in the decision whether or not to admit to hospital?

Task 4:

Write up a new FP10 for the patient

Pharmacy Stamp<br><br>Please don't stamp over age box	Age<br><br>D.O.B	Title Forename, Surname & Address
Number of day's treatment		
Endorsements		
Signature of Prescriber	Date	
For Dispenser No. of Prescns.	WORTOON CENTRAL PRIMARY CARE TRUST<br>Dr H Ferriman<br>HEALTH CENTRE<br>COUNCIL AVENUE	

NHS

015520000012

Task 3

What is the medical term for the violent shaking episodes?

What factors need to be considered when prescribing antibiotics to a patient in hospital?

Review the patient's drug kardex. Are the antibiotics prescribed correctly?

Task 5:

Write up the ciprofloxacin for discharge

SEND ALL COPIES TO PHARMACY **NuTH / IDL** 572001

Please use a ball point pen on a hard surface; please ensure all copies are legible

HOSP:..................... WARD:..............

INTERIM DISCHARGE LETTER

(A full Discharge Summary will be forwarded in due course)

G.P. Name:..

Address:..

....

**SCHEDULED DISCHARGE DATE** ....**TIME**.....................	
Surname	D.O.B..
First Names	Consultant
Address...	....
....	
Hosp. Reg. No.	

Dear Dr

Your patient was admitted under the care of ....

on...and discharged / transferred on the date above to ....

	DATE	CODE
Presenting Complaint/Diagnosis:..	....	....
....	....	....
Investigations:..	....	....
....	....	....
....	....	....
Treatment / Surgery..	....	....
....	....	....
Complications:..	....	....
....	....	....

DRAFT

Comments: ....

Follow up: .... at ....

**CURRENT MEDICATION (USE BLOCK LETTERS)** **Approved Name** **Form** **Dose/Frequency** PLEASE CANCEL THROUGH BOXES NOT USED	Planned duration of treatment / comments	Supply NOT reqd'	No. of days supply OR	No. of doses required	Treatment to be continued by GP	Pharmacy use: Quantity supplied
					YES / NO	
					YES / NO	
					YES / NO	
					YES / NO	
					YES / NO	
					YES / NO	
					YES / NO	
					YES / NO	
					YES / NO	
					YES / NO	

Medication on Admission No Longer Required / Other Comments Regarding Medication

Please check the compatibility of drugs supplied with ALL the patient's current medication

PRESCRIBER'S SIGNATURE: NAME (Print): Date: DESIGNATION: Dect No:	PIN No. (non-medical prescriber)	Initial here if Child Resistant Container is **NOT** required	Trans. by Date.	DISP. CHK.	Date

MAR/08

A teenager with a headache

Meningitis case

Task 1:

Which of these symptoms are you concerned about?

What are the medical terms for these symptoms?
What is your differential diagnosis at this point?

What would your next steps be?

What risk factors does Sophie have for your main differential diagnosis?

Task 2:

Use the clinical guidelines to prescribe the most appropriate antibiotic therapy on the drug kardex pr ovided.

How are you going to assess the effectiveness of the antibiotics?

Compare the clinical guidelines provided to the guidance in the BNF - why might different NHS Trusts use different guidelines?

Prescribe other therapy that could be of benefit for the patient in their clinical state.

Task 3:

Who do you think needs to be treated prophylactically and why?

What questions would you need to ask of each contact, before prescribing their medication?

Use the BNF to decide which antibiotic is the most appropriate and in what dose/duration for the pro phylaxis of *Neisseria meningitides*.

Significant Event Analysis: What can we learn from Mr Smith's case?

Introduction

In this 50 minute seminar you will work together to consider the events leading up to Mr Robert Smith's admission with acute sepsis (the same patient you met/ will meet in the SimMan session), using the technique of Significant Event Analysis/Audit (SEA) to identify positive and negative aspects of care and to learn from these. SEA is regularly used in primary care multidisciplinary teams, usually in the form of a monthly meeting to discuss 'significant events'. GPs must provide evidence of participation in SEA for appraisal and revalidation.

What is a significant event?

A common question often posed by primary care teams is: 'What exactly is a significant event'? The original definition by Pringle and colleagues (see below) is very broad. It is important to remember that a significant event can be either positive or negative. We can learn as much from good practice as from bad practice. Examples could range from a serious patient safety incident (for example, a medication error leading to death), to a moderate level error (for example, failure to act on laboratory findings resulting in a four-week delay in a diagnosis), to an event which demonstrates excellent care provision (for example, rapid diagnosis of unexpected malignancy in a fit young man), to one of a seemingly trivial nature which has serious administrative consequences (failing to change a recorded message on a Bank Holiday weekend).
Definition of a significant event: 'Any event thought by anyone in the team to be significant in the care of patients or the conduct of the practice.'
The interchangeable use of safety-related terminology (critical incident, error, near miss, adverse event and so on) by health professionals can cause confusion. All are 'significant events'. In one sense, because the definition of a significant event is all-embracing, this can make it easier for us to identify those issues where there are important learning opportunities for the team.

What is Significant Event Analysis/Audit?

Put simply, an SEA is a 'qualitative' method of clinical audit. In this respect it differs from the 'traditional' process of audit which most primary care teams will be familiar with: for example, when reviewing and improving care in the management of diabetes, asthma, IHD, or hypertension. These audits tend to deal with larger-scale 'quantifiable' patient data sets and involve defining criteria and setting standards which can be measured and compared against. However, SEA should involve a systematic attempt to investigate, review and learn from a single event that is deemed to be 'significant' by the healthcare team. Often, these types of 'significant events' will not be highlighted through 'normal' audit, but they still offer the primary care team valuable opportunities to improve the quality and safety of healthcare. An SEA provides us with a structured framework which can guide the primary care team when discussing and investigating a chosen significant event. Pringle's SEA definition: A process in which individual episodes (when there has been a significant occurrence either beneficial or deleterious) are analysed in a systematic and detailed way to ascertain what can be learnt about the overall quality of care, and to indicate any changes that might lead to future improvements.

Mr Robert Smith – simulated case – primary care information

CASE

D.O.B: 22/10/1945 **Age: 70 years**

Past medical history:

Diabetes type 2

Hypertension

Current medication:

Metformin 500mg three times daily

Gliclazide 160mg twice daily

Lisinopril 10mg daily

Aspirin 75mg daily

Atorvastatin 20mg daily

Allergy to penicillin (GI upset – documented in 1974)

Outstanding follow up: DNA podiatry appointment December 2015

Recent pathology and investigations:

16/02/2016 BP 156/96mmHg

Robert Smith<br>NHS: 404 631 555 678	68 Walker Gardens<br>Byker<br>Newcastle upon Tyne	Date of sample: 16/2/16

Sodium	135	(135-145mmol/L)
Potassium	3.6	(3.5-5mmol/L)
Creatinine	101	(70-150µmol/L)
Urea	6	(2.5-6.7mmol/L)
eGFR	67ml/min/1.73m2	
HbA1c	64mmol/mol (8%)	

Recent primary care consultations:

Dr Ferriman: 1/3/16

Hx: Seen in surgery. Painless small ulcer on left great toe noticed two weeks ago. Over past 4 days this has enlarged and become more painful. Patient concerned re: possible infection as has had similar previously.

Examination: Ulcer 1cm diameter dorsum of left great toe. Appears sloughy, surrounding skin hot and red, extending to 1st MTP. No tracking. Confirmed DP and PT pulses present.

Plan: As penicillin allergic for treatment with clarithromycin for 7 days and review INB at end of course.

Rx: Clarithromycin 500mg tablets 1 tablet PO BD for 7 days

Dr Jones: 6/3/16

Hx: Seen in surgery. Toe still sore and patient concerned it is becoming more swollen – struggling to get shoe on. Slow response to antibiotics. C/O nausea & abdominal discomfort, has missed some doses.

Examination: Left toe swollen, hot and red throughout, some slight yellow discharge from ulcer, no tracking past 1st MTP. Swab taken.

Plan: Switch to clindamycin due to side effects. Requested district nurse to visit for daily dressings and to ensure compliance with antibiotics.

Rx: Clindamycin 450mg BD for 7 days. Diarrhoea warning given.

Dr Jones: 8/3/16

Phone call from patient's son: prescription was received by pharmacy but not followed up by phone request for delivery and therefore has not been delivered – spoke to pharmacy– they will kindly deliver this afternoon.

Dr Ferriman: 11/3/16

Home visit: Visit requested by son, who is present. Son concerned re: drowsiness and apparent confusion since last night. Robert is lying in bed, drowsy but rousable.

Obvious confusional state – disorientated and keeps asking for wife who died 3 years ago.

Examination: Temp 38.2c, pulse 84bpm BP 160/94

Appears flushed and sweaty. Left foot red, hot and swollen to middle of dorsum of foot with some lymphatic tracking as far as ankle. Ulcer appears angry and red, now 1.5cm diameter and weeping thick yellow fluid. Left great toe swollen++.

Imp: Spreading cellulitis and likely sepsis

Plan: Arranged admission to EAU at RVI via urgent ambulance (within 1 hour). Son will travel with him to hospital. Advised to call 999 if any deterioration whilst awaiting ambulance.

Dr Ferriman: 11/3/16

Swab results obtained. Methicillin Sensitive Staphylococcus Aureus (MSSA) sensitive to:

Clarithromycin

Flucloxacillin

Clindamycin

Metronidazole

Ciprofloxacin

Significant Event Report
**Significant Event Analysis - Title:**
**Date of incident:**
**Date of meeting when event was discussed – this can be in your practice or in a self-directed learning group.**
**Description of event - what actually happened and what was your direct involvement in the event?**
**What went well?**<br><br>**And what could have been done better?**
**What were the learning outcomes?**<br><br>**For yourself**

For the practice [if appropriate]

What changes have been agreed?
This should clearly identify who is responsible for each action and a date that the action is to be completed. The initials and job title can be used to maintain confidentiality.

For yourself

For the practice [if appropriate]

How and when will the effect of these changes be reviewed?

SimMan sepsis: Mr Robert Smith

Mr Robert Smith has been admitted to hospital via his GP. In this 1 hour simulated case session, you will be working in inter-professional teams to solve clinical problems related to Mr Smith's admission.

11/03/2016:

Dear Dr,

Robert Smith, 70-yr-old male, 68 Walker Gardens, Byker, Tyne & Wear Please accept this patient for admission, found by son with confusion, feverish. Currently on treatment for diabetic foot ulcer- had 5/7 clarithromycin, switched to clindamycin 3/7 ago.

PMH: Diabetes (type II), Hypertension

Meds: Metformin, Gliclazide, Lisinopril, Aspirin, Atorvastatin. Penicillin allergic.

??Sepsis

Regards,

Dr H Ferriman

Additional Resources: https://www.england.nhs.uk/wp-content/uploads/2014/09/psa-sepsis.pdf **http://www.pharmaceutical-journal.com/learning/learning-article/causes-and-features-of-sepsis/10975342.article**

Mr Robert Smith – Simulated Case – ON ADMISSION

Mr Robert Smith has been admitted to the emergency assessment unit and has been triaged by the nurse practitioner on duty.

- NEWS scoring carried out (see attached chart).
- As a result of the NEWS score the junior doctor is called to assess the patient.
- Patient is speaking but not in full sentences

1) What would you do first?

A Examine the toe wound
B Administer oxygen
C Administer paracetamol
D Obtain IV access

2) Does he meet any of the SIRS criteria? If so, which?

3) What information do you need to decide on the next antibiotic?

3) Which antibiotic/s would you administer?

A Cefuroxime plus metronidazole
B Amoxicillin plus ciprofloxacin
C Tazocin
D Chloramphenicol

4) Write up the antibiotic/s on the medicine chart provided – consider the dose and the route of administration.

On Admission: Bloods

Robert Smith	68 Walker Gardens Byker<br>Tyne and Wear	Date of sample: 11/3/16
NHS: 404 631 555 678		

Sodium 135 (135-145mmol/L)

Potassium 4.6 (3.5-5mmol/L)

Creatinine 180 (70-150µmol/L)

Urea 12 (2.5-6.7mmol/L)

eGFR 35ml/min/1.73m^2

WCC 13 x 10^9/L (4-11 x 10^9/L)

Neutrophils 8 x 10^9/L (2-7.5 x 10^9/L)

Platelets 120 x 10^9/L (150-400 x 10^9/L)

Alanine transaminase (ALT) 28 (3-35iu/L)

Aspartate transaminase (AST) 28 (3-35iu/L)

Alkaline phosphatase (ALP) 150 (30-300iu/L)

Total bilirubin (TBIL) 9 (3-17µmol/L)

Lactate 7 (< 2mmol/L)

Random blood glucose 9mmol/L

Clotting screen = NAD

Blood culture = Awaiting results

Hospital **Chart No.**

CODES FOR NON-ADMINISTRATION OF

PRESCRIBED MEDICINE

In situations where a does of a medicine is not administered and the matter cannot be resolved immediately the nurse must:-

a) Record on the medicine chart the appropriate code number for the reason why the dose was not administered and initial this code.

b) **TAKE APPROPRIATE ACTION** to resolve the matter **PROMPTLY** so that patient treatment is not compromised.

For further details refer to the 'Purple Booklet'

Patient refuses	1	Unable to swallow	8
Patient not present on ward	2	Vomiting/nausea	9
Medicine not available	3	Time varied on Dr's instructions	10
(obtain as soon as practicable)		Once only/PRN medication given	11
Instructions not clear or legal	4		
Patient Self-Administered Medicine	5		
Nil by mouth	6		
Asleep/drowsy	7		

To be specified in the Nursing Care Plan

Possible drug reaction/ side effect	12
Other reasons	13

Name of Patient:
Patient Number:
Ward
DOB
Date of Admission
Consultant
Weight Surface Area

DRUG SENSITIVITIES **(drugs must not be administered unless this box has been completed. List drugs or mark 'none known')**

ONCE ONLY

Date	Time	Pharmacy	Drug (Approved Name)	Dose	Route	**Prescriber's Signature** NAME	**Time** Given	**Given** by	**Checked** by

OTHER CHARTS IN USE

Date	Type of Chart	Details	Signature NAME

OTHER DETAILS

Drug History Taken By:	Date:
Drug Chart Written By:	Date:
Discharge Prescription Written By:	Date:

Name Number

REGULAR THERAPY

Date												

DRUG (Approved Name)																
				0800												
Date	Dose	Route	Notes	1200												
				1800												
Prescribers Signature		Duration of therapy		2200												
Date Stopped	Initials	Pharmacy use														

DRUG (Approved Name)																
				0800												
Date	Dose	Route	Notes	1200												
				1800												
Prescribers Signature		Duration of therapy		2200												
Date Stopped	Initials	Pharmacy use														

DRUG (Approved Name)																
				0800												
Date	Dose	Route	Notes	1200												
				1800												
Prescribers Signature		Duration of therapy		2200												
Date Stopped	Initials	Pharmacy use														

DRUG (Approved Name)																
				0800												
Date	Dose	Route	Notes	1200												
				1800												
Prescribers Signature		Duration of therapy		2000												
Date Stopped	Initials	Pharmacy use														

DRUG (Approved Name)																
				0800												
Date	Dose	Route	Notes	1400												
				1800												
Prescribers Signature		Duration of therapy		2200												
Date Stopped	Initials	Pharmacy use														

DRUG (Approved Name)																
				0800												
Date	Dose	Route	Notes	1200												
				1800												
Prescribers Signature		Duration of		2200												
Date Stopped	Initials	Pharmacy use														

DRUG (Approved Name)																
				0800												
Date	Dose	Route	Notes	1200												
				1800												
Prescribers Signature		Duration of		2200												
Date Stopped	Initials	Pharmacy use														

DRUG (Approved Name)																
				0800												
Date	Dose	Route	Notes	1200												
				1800												
Prescribers Signature		Duration of		2200												
Date Stopped	Initials	Pharmacy use														

VARIABLE DOSE MEDICATION

DRUG (Approved Name)					Route / Pharmacy	
Date	INR§	Dose	Prescribed By	Name	Given By	Pharmacy

DRUG (Approved Name)					Route / Pharmacy	
Date	INR*	Dose	Prescribed By	Name	Given By	Pharmacy

DRUG (Approved Name)					Route / Pharmacy	
Date	INR*	Dose	Prescribed By	Name	Given By	Pharmacy

DRUG (Approved Name)					Route / Pharmacy	
Date	INR*	Dose	Prescribed By	Name	Given By	Pharmacy

§ To be used for recording INR in patients taking oral anticoagulants

AS REQUIRED THERAPY

Drug (Approved Name)			Date												
			Time												
Dose	Route	Start Date	Dose / Route												
			Given												
Frequency & Instructions		Signature	Date												
		Name	Time												
Stop Date	Initials	Pharmacy	Dose / Route												
			Given												

Drug (Approved Name)			Date												
			Time												
Dose	Route	Start Date	Dose / Route												
			Given												
Frequency & Instructions		Signature	Date												
		Name	Time												
Stop Date	Initials	Pharmacy	Dose / Route												
			Given												

Drug (Approved Name)			Date												
			Time												
Dose	Route	Start Date	Dose / Route												
			Given												
Frequency & Instructions		Signature	Date												
		Name	Time												
Stop Date	Initials	Pharmacy	Dose / Route												
			Given												

Antibiotic guidance (septicaemia and severe infections)

This guidance has been extracted from the Newcastle upon Tyne Hospital NHS Foundation Trust Antimicrobial Handbook, Feb 2016.

SEPTICAEMIA AND SEVERE INFECTIONS

Septicaemia or severe infection of unknown cause

1. Septicaemia is defined by the British Society for Antimicrobial Chemotherapy as: 'The presence of large numbers of bacteria in the bloodstream, often associated with systemic signs and symptoms such as fever, rigors and headache.' See the BSAC website for more details.

2. Systemic Inflammatory Response Syndrome (SIRS)

TWO or more of:

Temperature >38ºC or <36ºC
Heart rate > 90 bpm
Respiratory rate >20 breaths/min
WBC >12 or <4 (x109/L)

Sepsis is defined as **SIRS + confirmed infection or clinical evidence of infection**

Severe sepsis is defined as
sepsis + organ dysfunction: hypotension or organ hypoperfusion

3. In seriously ill patients, consult a microbiologist for advice on treatment.

4. It is important to realise that a broad spectrum combination may still have gaps (MRSA, for example) or may provide less than optimal therapy against some organisms (e.g. Pseudomonas). No agent or combination of agents can hope to cover all eventualities. This recommendation is necessarily a compromise; the patient should be examined thoroughly for a source of infection and therapy tailored to counter likely pathogens (see other sections on individual infections). If in doubt, consult a microbiologist.

5. Blood cultures are essential before starting treatment; therapy must be reviewed when results are available or if the patient fails to respond within 24-48 hours.

6. It is unusual for a septicaemia to be of totally unknown origin. The patient's history will often allow a good guess as to likely infecting organisms to be made. This is frequently not the case in neutropenic patients.

7. Coagulase-negative staphylococci (sometimes reported as Staph. epidermidis) are of low pathogenicity and are most commonly encountered as a cause of septicaemia when associated with an intravenous cannula. In this case, consider removing the cannula as infection may be difficult to eradicate in its presence.

8. These recommendations do not apply to sepsis in neutropenic patients. Units which deal regularly with these patients should have their own antibiotic policies for sepsis in neutropenia and these should be followed. Separate guidelines for staff who do not routinely manage neutropenic patients but who see such patients in the course of their usual work can be found in 'Management of sepsis in neutropenic patients.'

Likely pathogens: Very varied. Mixed Gram-positive and Gram-negative infections may occur.

Recommended antibiotics:

First choice:	Cefuroxime plus metronidazole
Second choice:	Amoxicillin plus ciprofloxacin (plus metronidazole if anaerobes are suspected
**Care of the Elderly Wards and patients >65yrs on Acute Medical Wards and A&E.**	
First choice	Piperacillin - tazobactam ('Tazocin') 2.25g - 4.5g QDS
Alternatives	For patients with history of penicillin hypersensitivity.<br><br>MUST discuss with senior clinician or microbiology.<br><br>IV Chloramphenicol* 12.5mg/kg QDS

Length of treatment: for confirmed sepsis, a minimum of 7 days, but this may vary according to clinical circumstances.

*Chloramphenicol is only indicated for life threatening infections, due to the risk of blood dyscrasias, a FBC MUST be taken at initiation and throughout the treatment course. Prolonged >14days and repeated courses should be avoided. Chloramphenicol should be avoided in patients with pre-existing myelosupression

SEVERE SEPSIS SCREENING TOOL

Use the screening tool in all adult patients who are found to have an infection or trigger on the EWS 3 or more

SIRS
2 or more criteria met?

- ☐ Temp > 38.3°C
- ☐ Heart rate > 90/min
- ☐ WCC > 12 x10^9/l
- ☐ Temp < 36°C
- ☐ Respiratory rate rate > 20/min
- ☐ WCC < 4 x10^9/l

If patient is* ***neutropenic* *inform senior doctor and follow* ***neutropenic sepsis guidelines***

☐ **YES**

☐ **NO** → **Follow standard track & trigger protocol**
Reapply screening tool if situation changes

INFECTION
Suspected or proven

- ☐ Pneumonia or LRTI
- ☐ Intraperitoneal infection
- ☐ Bone or joint infection
- ☐ Endocarditis
- ☐ Foreign body/ implanted device infection
- ☐ Skin or soft tissue infection *(incl. wound)*
- ☐ Urinary tract infection
- ☐ Biliary tract infection
- ☐ ENT/ maxillofacial infection
- ☐ CNS infection
- ☐ Bloodstream catheter infection
- ☐ Female reproductive tract infection

☐ **YES**

☐ **NO** → **Patient has SIRS, consider other causes**: pancreatitis, burns, trauma, transfusion reaction, thromboembolism
Follow standard track & trigger protocol

SEPSIS

SEPSIS = Infection + 2 or more SIRS criteria

- ☐ Clinical assessment and management (ABC approach)
- ☐ Take appropriate microbiological samples
- ☐ Give antibiotics as per local guidelines
- ☐ Screen for SEVERE SEPSIS (see below)

SIGNS OF ORGAN DYSFUNCTION

SEVERE SEPSIS

SEVERE SEPSIS = SEPSIS + 1 SIGN OF ORGAN DYSFUNCTION

- ☐ SBP < 90mmHg or MAP < 65 mmHg
- ☐ New need for oxygen to keep SpO_2 >90%
- ☐ INR > 1.5 or APTT > 60s
- ☐ Urine Output < 0.5ml/kg/hr for 2 hours
- ☐ Lactate > 2mmol/l
- ☐ Platelets < 100 x 10^9/l
- ☐ Bilirubin > 34 µmol/l
- ☐ Creatinine >177 µmol/l

☐ **YES**

☐ **NO** → Manage as **SEPSIS** as above
Reassess for **SEVERE SEPSIS** if patient deteriorates

SEVERE SEPSIS criteria met
TIME ZERO = ___:___ (24 h clock)
Complete **SEPSIS SIX** within1 hour

SEVERE SEPSIS MANAGEMENT PATHWAY

If patient diagnosed of **SEVERE SEPSIS** please complete the **Sepsis Six** within one hour of meeting criteria **(TIME ZERO)**
Document any limitations of treatment as appropriate

**SEPSIS SIX©**	**Time**	**Initial**	**Relevant result (Reason if not done)**
1. **Oxygen**: high flow 15 l/min via non-rebreathe mask<br>Target saturation > 94%			
2. **Blood cultures:** take at least one set plus all relevant blood tests, i.e., FBC, U&Es, LFT, clotting, glucose, lactate, other cultures as appropriate			
3. **IV antibiotics:** as per Trust guidelines			
4. **Fluid resuscitate:**<br>if $BP_{systolic}$ < 90 give boluses of Hartmann's or 0.9% saline 20 mls/Kg			
5. **Check lactate**<br>if lactate > 2 mmol/l give 20 mls/kg of Hartmann's or 0.9% saline			
6. Commence fluid balance and consider urinary catheterisation			

Plus

Discuss with **Senior Doctor**<br>Inform **Critical Care** if appropriate			

If persisting signs of organ dysfunction follow with the resuscitation bundle

**RESUSCITATION BUNDLE**	**Time**	**Initial**	**Relevant result (Reason if not done)**
Ensure **Critical Care** attends urgently (if not already). Transfer to Critical Care area			
Ensure patient has received adequate **fluid resuscitation**: boluses of 20 mls/kg of Hartman's or 0.9% saline up to a max of 60 mls/Kg			
If still shocked (low BP/ urine output/ high lactate) **insert central venous catheter**			
Aim to achieve a **CVP of 8 – 12 mmHg** with fluid boluses			
Take heparinised sample of blood from central line (use ABG syringe) and check **$ScvO_2$ > 70%**			
Ensure **Hb > 7 g/dl**, consider transfusion if necessary			
Consider **noradrenaline** if still shocked after fluid boluses or **dobutamine** if **$ScvO_2$** < 70%			

The Newcastle upon Tyne Hospitals NHS
NHS Foundation Trust

Newcastle Upon Tyne Hospitals Adult NEWS Chart

Please Affix patient identification label in box below and document date chart started

Date DD/MM/YYYY

Surname	
Forename	
Patient i.d.No.	
D.O.B. DD/MM/YYYY	

Outline Clinical Response to NEWS Triggers

NEWS SCORE	MINIMUM FREQUENCY OF MONITORING	CLINICAL RESPONSE
0	12 hourly observations	• Continue routine NEWS monitoring with every set of observations
Total: 1-4	4 hourly observations	• Inform registered nurse who must assess the patient;<br>• Registered nurse to decide if increased frequency of monitoring and / or escalation of clinical care is required
Total: 5 or more or 3 in one parameter	1 hourly observations	• NEWS responder<br>• Response time 30 mins
Total: 7 or more<br>Or 3 in two parameters	Continuous monitoring	• Senior NEWS responder<br>• AND Outreach<br>• Response time 10 mins<br>Outreach<br>☎ 48817 FH<br>☎ 48881<br>☎ 29995 RVI<br>☎ 23956

Oxygen Prescription (circle target)

target saturation 94-98% (air ↔ 2 L/min ↔ 4 L/min ↔ 10-15 L/min)

target saturation 88-92% (air ↔ 24% ↔ 28% ↔ 35% venturi)

other target specify ____% **delivery method** ____________

PCA continuous oxygen ☐ **use minimum O_2 to achieve target**

prescriber/ transcriber date D D M M Y Y Y Y **initial time** H H M M

Escalation Policy

Registered nurse assesment
↓
Ward NEWS Responder
↓
Senior Ward NEWS Responder
↓
Consultant
↓
Critical Care
☎ 48483 Anaesthetic 2nd call FH
☎ 48812 GITU resident FH
☎ 48830 Cardiac ICU 2nd call FH
☎ 29999 Critical Care RVI

CALLING OUTREACH IS <u>NOT</u> A CRITICAL CARE REFERRAL.

SENIOR WARD MEDICAL STAFF MUST DIRECTLY CALL ICU TEAM

STOP! THINK! Why has my Patient triggered?

National Early Warning Score (NEWS)

Risk of Hypercapnic Respiratory Failure	
COPD	
Obesity/ Hypoventilation	
Other	
Sign & date variance box	

PHYSIOLOGICAL PARAMETERS	3	2	1	0	1	2	3	Variance	Date & Time	Sign	Temp Perm
Respiration Rate	≤8		9-11	12-20		21-24	≥25				T P
Oxygen Saturations	≤91	92-93		≥94							T P
Oxygen Saturations (Risk of Hypercapnic Respiratory Failure)	≤79	80-84	85-87	≥88	Risk of Hypercapnic Respiratory Failure						T P
Any Supplemental Oxygen		Yes		No							T P
Temperature	≤35.0		35.1-35.9	36.0-38.0	38.1-38.9	≥39.0					T P
Systolic BP	≤90	91-100	101-110	111-179	180-199		≥200				T P
Heart Rate	≤40		41-50	51-90	91-110	111-129	≥130				T P
Level of Consciousness	**NEW** agitation or confusion score 3 X			A			V,P, or U				T P
Urine Output	<30ml/hr				Patients with established chronic/ anuric renal failure score 0						T P
Nursing Concern	Yes			No	Confirm with nurse in charge						T P

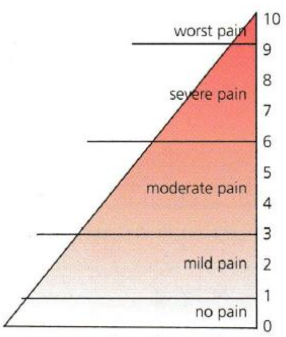

Scores persistently 7 or above contact Pain Team

NEWS Clinical Response

Date	Time	NEWS Score	Individual Called		Sign Ward Staff	Response	Time	Sign NEWS Responder
			Name	Grade				

Please check variance before scoring. Document number in top or bottom boxes

Parameter		Score	
	DATE		DATE
	TIME		TIME
RESP. RATE	≥25	3	≥25
	21-24	2	21-24
	12-20		12-20
	9-11	1	9-11
	≤8	3	≤8
SpO_2	≥94		≥94
	92-93	2	92-93
	≤91	3	≤91
Inspired O_2	%/L	2	%/L
TEMP	≥39°	2	≥39°
	38°	1	38°
	37°		37°
	36°		36°
	≤35°	1	≤35°
		3	
NEW SCORE uses Systolic BP	≥220	3	≥220
	210	3	210
	200	3	200
	190	1	190
	180	1	180
	170		170
	160		160
	150		150
	140		140
	130		130
	120		120
	70	3	70
	60		60
	50		50
HEART RATE	≥130	3	≥130
	120	2	120
	110		110
	100	1	100
	90		90
	80		80
	70		70
	60		60
	50		50
	40	1	40
	30	3	30
Level of Consciousness	Alert		Alert
	V / P / U	3	V / P / U
Urine Output <30ml/hr	Y/N	3	Urine Output
Nursing Concern	Y/N	3	Concern
TOTAL NEW SCORE			TOTAL SCORE
	Initials		Initials
Additional Parameters	Pain Score		Pain Score
Monitoring Frequency			Monitor Freq
News Escalation Plan Y/N n/a			Escal Plan
RN Review Initials			RN Initials

NEWS Clinical Response

Date	Time	NEWS Score	Individual Called		Sign Ward Staff	Response	Time	Sign NEWS Responder
			Name	Grade				

Potts Print (UK). Nov 2015

Medical Protection

pda the pharmacists' defence association

CAIPE

Centre For The Advancement Of Interprofessional Education

BRITISH PHARMACOLOGICAL SOCIETY

NPA National Pharmacy Association

United Kingdom Clinical Pharmacy Association
