# Supplementary material for: Designing and evaluating an interprofessional education conference approach to antimicrobial education
Source: BMC Med Educ. 2020 Oct 13;20:360. doi: 10.1186/s12909-020-02252-9 (PMC7552509; doi:10.1186/s12909-020-02252-9)
Supplement: Supplementary file 2 — Additional file 2. Facilitators and barriers to learning in each workshop. Thematic analysis of student free text responses to questions about facilitators and barriers to learning in each workshop. The table shows broad themes that arose from the data analysis, details about what each theme encompasses, and the number of responses within each theme by medical and pharmacy students. Abbreviations: MCQs, Multiple Choice Questions. [file 12909_2020_2252_MOESM2_ESM.docx]

### Supplementary File 2: Facilitators and barriers to learning in each workshop

| **Theme** | **Encompasses** | **1. Choosing the right Antibiotic**  **Medicine  Pharmacy** | | **2. Significant Event Audit**  **Medicine  Pharmacy** | | **3. SimMan**  **Medicine  Pharmacy** | |
| --- | --- | --- | --- | --- | --- | --- | --- |
| ***Give examples of what you found interesting or helpful*** | | | | | | | |
| Learning techniques | Acronyms, cases, terminology etc. | 23 | 13 | 5 | 6 | 2 | 8 |
| Practical skills | Prescribing, interpreting lab results etc. | 37 | 19 | 16 | 10 | 2 | 12 |
| Understanding & Reasoning | Roles of others. Why an approach is taken e.g. ABCDE | 5 | 2 | 25 | 17 | 2 | 7 |
| Teamwork | Skill of working with others | 19 | 19 | 3 | 5 | 12 | 14 |
| Teaching resources | SimMan, MCQs, workbook, videos etc. | 5 | 1 | 27 | 8 | 47 | 23 |
| Teaching techniques | Role play, facilitation etc. | 19 | 8 | 4 | 2 | 50 | 49 |
| Logistics | Group size, room size etc. | 0 | 1 | 0 | 0 | 1 | 1 |
| Reflection | Importance/value of interdisciplinary learning | 14 | 6 | 4 | 16 | 8 | 11 |
| NA/No | - | 1 | 3 | 2 | 3 | 1 | 0 |
| No answer provided |  | 22 | 61 | 31 | 68 | 11 | 33 |
| ***What specific aspects hindered learning and how could they be addressed?*** | | | | | | | |
| Logistics | Timing, room size, group size etc. | 14 | 9 | 7 | 15 | 19 | 25 |
| Educational content | Lack of sufficient prior knowledge, topic accessibility etc. | 5 | 15 | 13 | 9 | 4 | 12 |
| Teamwork | Lack of co-operation, not enough medical students etc. | 7 | 17 | 8 | 6 | 1 | 4 |
| Utilisation of resources | Use SimMan more, more MCQs etc. | 16 | 3 | 5 | 6 | 25 | 10 |
| Personal factors | Sleepiness, having to leave early etc. | 1 | 0 | 0 | 3 | 0 | 0 |
| Reflection | Importance/value of training | 0 | 0 | 0 | 2 | 0 | 0 |
| NA/No | - | 8 | 26 | 11 | 20 | 16 | 21 |
| No answer provided | - | 49 | 66 | 51 | 71 | 25 | 56 |

**Supplementary File 2 legend**: Thematic analysis of student free text responses to questions about facilitators and barriers to learning in each workshop. The table shows broad themes that arose from the data analysis, details about what each theme encompasses, and the number of responses within each theme by medical and pharmacy students. Abbreviations: MCQs, Multiple Choice Questions.
